# Preservation of *Bacillus subtilis*’ cellular liquid state at deep sub-zero temperatures in perchlorate brines

**DOI:** 10.1038/s42003-024-06277-4

**Published:** 2024-05-16

**Authors:** Stewart Gault, Fernanda Fonseca, Charles S. Cockell

**Affiliations:** 1 UK Centre for Astrobiology, SUPA School of Physics and Astronomy, University of Edinburgh, James Clerk Maxwell Building, Peter Guthrie Tait Road, Edinburgh, EH9 3FD UK; 2 Université Paris-Saclay, INRAE, AgroParisTech, UMR SayFood, F-91120 Palaiseau, France

**Keywords:** Bacteria, Biophysics

## Abstract

Although a low temperature limit for life has not been established, it is thought that there exists a physical limit imposed by the onset of intracellular vitrification, typically occurring at ~−20 °C for unicellular organisms. Here, we show, through differential scanning calorimetry, that molar concentrations of magnesium perchlorate can depress the intracellular vitrification point of *Bacillus subtilis* cells to temperatures much lower than those previously reported. At 2.5 M Mg(ClO_4_)_2_, the peak vitrification temperature was lowered to −83 °C. Our results show that inorganic eutectic salts can in principle maintain liquid water in cells at much lower temperatures than those previously claimed as a lower limit to life, raising the prospects of active biochemical processes in low temperature natural settings. Our results may have implications for the habitability of Mars, where perchlorate salts are pervasive and potentially other terrestrial and extraterrestrial, cryosphere environments.

## Abstract (web summary)

A differential scanning calorimetry study of *Bacillus subtilis* vitrification shows that perchlorate salts can lower the onset of intracellular vitrification, with implications for the low temperature limit of life in salt-rich subzero environments.

## Introduction

What defines the limits to life in extreme environments? While the limit to life in high temperature environments is well characterized^1^, the limit imposed by cold environments is less well established. It has been argued that there should be no fundamental low temperature limit to life as theoretical and experimental repair rates of cells exceed the spontaneous rate of cellular damage^2^. However, this argument could not be reconciled with the fact that in vivo microbial metabolism lower than −23 °C was difficult to observe.

Clarke et al.^3^, argued that while life may not exhibit a thermodynamic limit at low temperatures, it does experience a physical limit enforced by the onset of intracellular vitrification. Intracellular vitrification occurs when a cell’s extracellular environment begins to freeze, and extracellular solutes become concentrated, generating an osmotic gradient across the cell membrane, causing water to leave the cell. This process of freezing, solute concentration, and water loss continues until a glass transition occurs (intracellular vitrification), accompanied by a large increase in cellular viscosity^4^. Consequently, biochemical reactions cease. Clarke et al., found that the onset of intracellular vitrification occurred around −20 °C for assayed unicellular organisms^3^, which coincides with the lowest reported values of cellular metabolic activity such as those of *Planococcus halocryophilus*^5^. Sformo et al., have reported mean intracellular vitrification temperatures of −71 °C for *Cucujus clavipes puniceus* larvae^6^. The larval freeze resistance and low vitrification temperature were potentially caused by high intracellular glycerol concentrations and the expression of antifreeze proteins. Furthermore, it has also been demonstrated that the exogenous addition of the cryoprotectants glycerol and dimethyl sulfoxide (DMSO) depressed the vitrification point of *Lactobacillus delbrueckii* ssp. *bulgaricus* CFL1 to −45 and −50 °C respectively^7^.

Determining what fundamentally establishes the low temperature limit for life is essential in evaluating the habitability of low temperature environments beyond Earth. Such low temperature environments are represented by the Martian near and deep subsurface^8–12^, as well as the subsurface oceans of Europa, Enceladus, and Titan^13–16^. With regards to Mars, multiple studies have considered its potential to host aqueous environments^9,10^. Putative Martian aqueous environments are thought to experience temperatures lower than −70 °C, therefore these environments must contain a substance which can significantly depress the freezing point of water. To allow a liquid state to persist at these low temperatures these environments have been theorized to contain high concentrations of perchlorate salts, such as Mg(ClO_4_)_2_ and Ca(ClO_4_)_2_, due to their extremely low eutectic temperatures^17,18^ and their detection on the Martian surface^19^. The potential for aqueous, perchlorate-rich environments raises the question of whether extant life could be found in such an environment, or whether it is possible to inadvertently contaminate such an environment with viable terrestrial life. Our previous research sought to understand the potential for biochemistry in these environments. We found that while perchlorate salts lower the activity of enzymes, high hydrostatic pressures can rescue this activity^20^, and that perchlorate salts can increase enzyme activity at low temperatures^21^. However, these results are only relevant when biomacromolecules are able to freely diffuse and execute their biological functions, and at −70 °C one would expect any life to be frozen and incapable of biochemistry.

Therefore the question arose as to whether deeply eutectic salts, such as perchlorates, can depress intracellular vitrification in lieu of evolutionary biological adaptations. If so, this would demonstrate that abiotic environmental parameters can affect the low temperature limit for life.

Here, we investigated the intracellular vitrification of *Bacillus subtilis* (*B. subtilis*) cells exposed to increasing magnesium perchlorate concentrations with differential scanning calorimetry (DSC). As 0.3 M Mg(ClO_4_)_2_ was previously determined to be the upper growth limit for *B*. subtilis^22^, all cells exposed to Mg(ClO_4_)_2_ concentrations greater than 0.3 M were taken to be dead but not lysed. We show that 2.5 M Mg(ClO_4_)_2_ can lower the vitrification point of *B. subtilis* cells to temperatures lower than those previously reported in cells and down to temperatures associated with many perchlorate-containing environments on Mars.

## Results

### Cellular vitrification

**Fig. 1:**
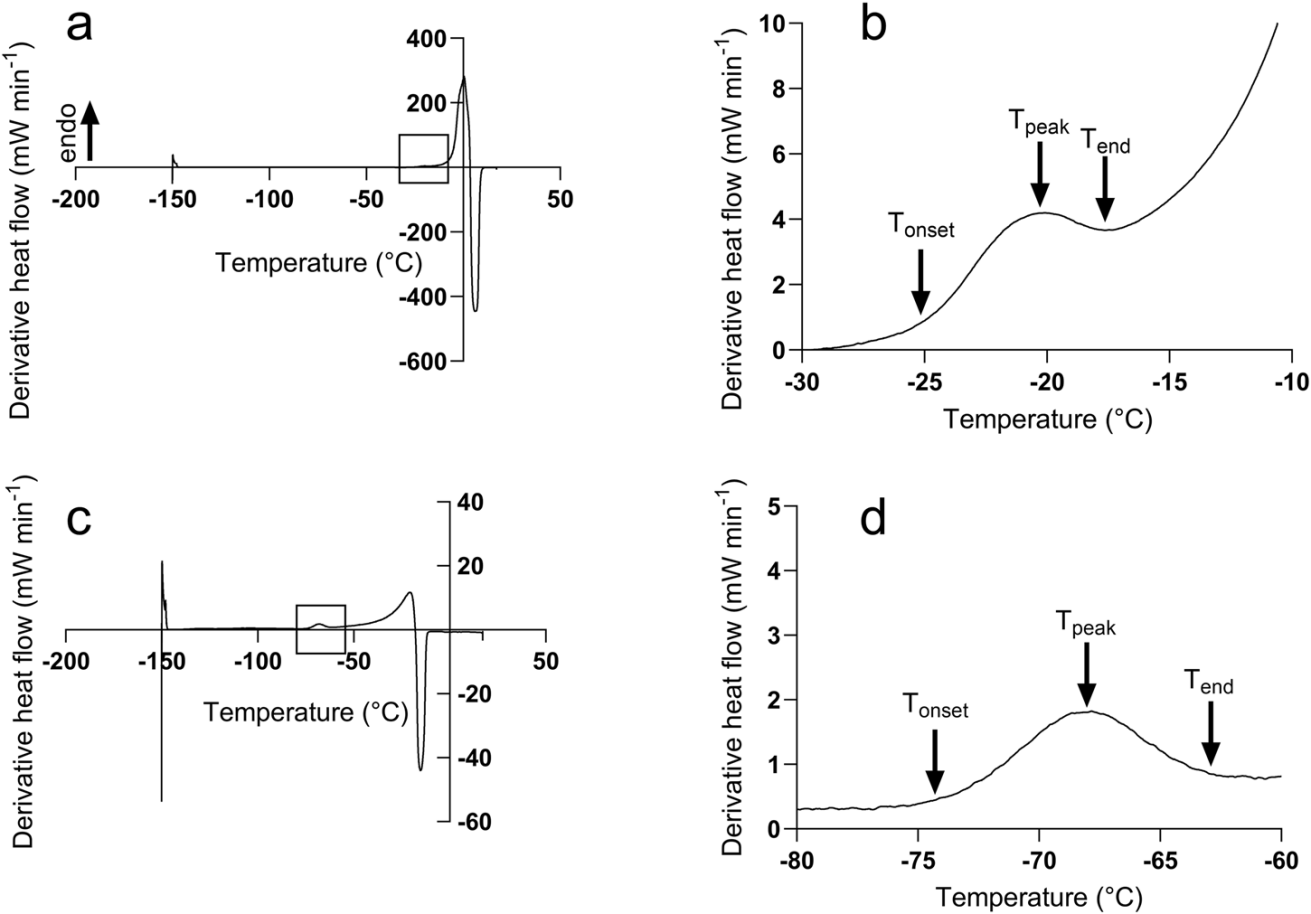
Representative first derivative of DSC thermograms of *Bacillus subtilis* cells across Mg(ClO_4_)_2_ concentrations. The first derivative of DSC thermograms of *Bacillus subtilis* cells during the heating ramp in **a** water washed cells, **c** cells exposed to 2 M Mg(ClO_4_)_2_, with the location of the vitrification peaks denoted by the black boxes. Panels **b** and **d** show representative rescaled thermograms of **a** and **c** revealing the vitrification peaks. Panels **b** and **d** show the recorded features of the vitrification signal, the T_onset_, T_peak_, and T_end_.

Figure 1a, c shows representative first derivatives of the DSC heating scan traces of *B. subtilis* in deionized water and 2 M Mg(ClO_4_)_2_, respectively. The most prominent feature of each scan is the melting of ice in the sample. Magnesium perchlorate’s ability to depress the freezing point of water is evidenced by the gradual leftward trend of the melting transition with increasing perchlorate concentration. Additionally, of note is the gradual decrease in energy of the melting transition with increasing perchlorate concentration. Due to the large melting peak signal, the cellular vitrification signal is partially masked by the axis scale, therefore Fig. 1b, d shows a rescaled inset (denoted by black squares) from Fig. 1a, c respectively, revealing the intracellular vitrification signal. The initial upwards inflection of the vitrification signal was recorded as the T_onset_, then the peak as T_peak_, and where the signal finishes was recorded as T_end_. This order was because the vitrification signal is observed during the heating ramp, whereas in a natural environment vitrification would occur during cooling, and so these events would be technically reversed.

**Fig. 2:**
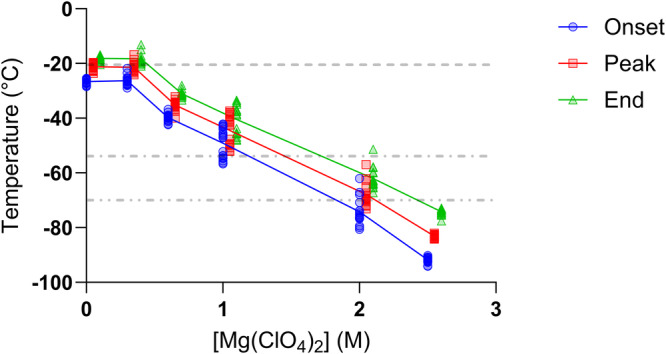
Glass transition temperatures of *Bacillus subtilis.* The onset (blue circles), peak (red squares), and end (green triangles) temperature of the *Bacillus subtilis* intracellular glass transition across Mg(ClO_4_)_2_ concentrations. The peak and end values have been offset by 0.05 and 0.1 respectively for visual clarity. Dashed lines denote mean vitrification temperatures from previous literature, Clarke et al., 2013 (- - -)^3^, Fonseca et al., 2016 (- • -)^7^, Sformo et al., 2010 (- • •)^6^. Number of replicates, 0 M: 17, 0.3 M: 16, 0.6 M: 20, 1.0 M: 18, 2.0 M: 16, 2.5 M: 16.

Figure 2 shows the recorded onset, peak, and end of intracellular vitrification across the range of assayed Mg(ClO_4_)_2_ concentrations. In the absence of Mg(ClO_4_)_2_, *B. subtilis* exhibited a mean T_peak_ of −21.2 °C, with 0.3 M Mg(ClO_4_)_2_ having little effect on T_peak_ as it remained at −21.4 °C. At higher assayed Mg(ClO_4_)_2_ concentrations, the T_peak_ of *B. subtilis* decreased in a near linear concentration-dependent manner. A concentration of 0.6 M Mg(ClO_4_)_2_ caused T_peak_ to decrease to a mean of −35.2 °C, with 1, 2, and 2.5 M reducing the mean T_peak_ to −44.5 °C, −67.9 °C, and −83.2 °C respectively. The dashed lines in Fig. 2 demonstrate how the observed intracellular vitrification temperatures compare to those observed in previous literature.

### Cellular lipid transition

**Fig. 3:**
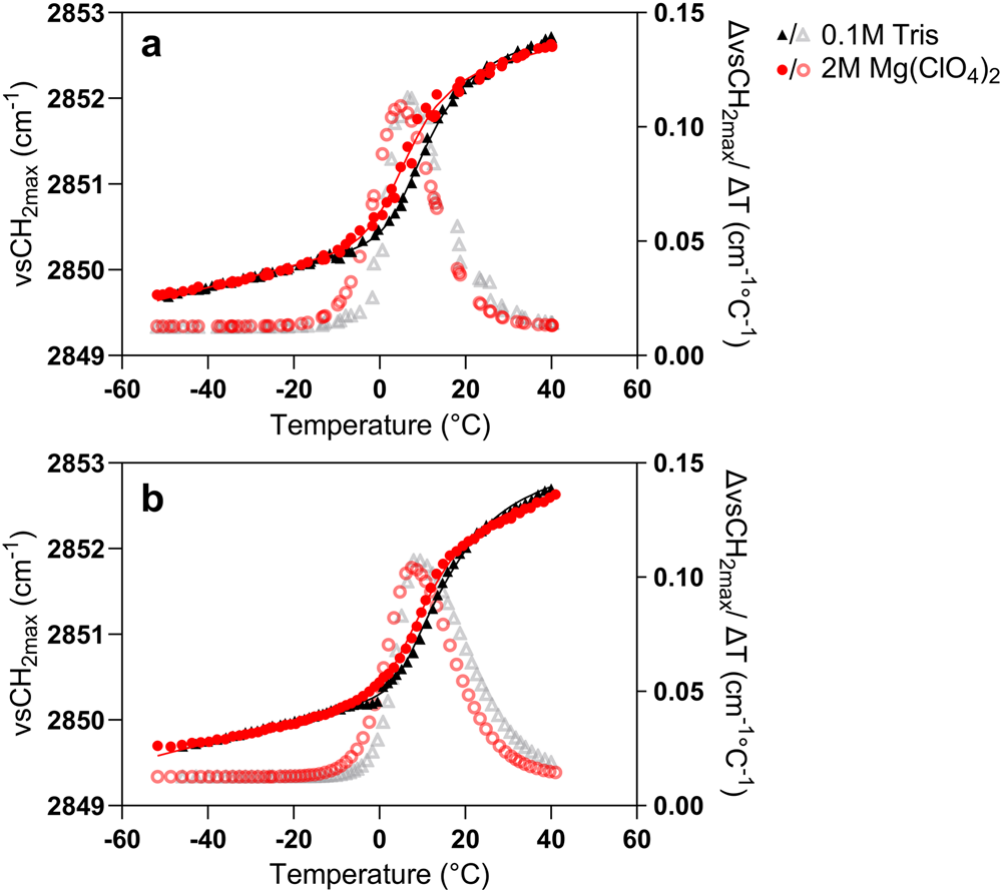
Lipid transition of *Bacillus subtilis* cells. Membrane lipid phase behavior (peak position of the symmetric CH_2_ stretching vibration band, *ν*_s_CH_2_) during cooling (**a**) and heating (**b**) of *Bacillus subtilis* cells in the presence of Mg(ClO_4_)_2_ (red circles and continuous line) and in Tris buffer (black/ gray triangles and continuous line). Hollow symbols indicate the respective first derivative of the *ν*_s_CH_2_ band and the maximum of each one corresponds to the lipid transition temperature of membrane solidification, T_s_, (**a**) and membrane melting, T_m_ (**b**).

The organization of the membrane lipids was evaluated during freezing and heating by FTIR spectroscopy and is presented in Fig. 3. The observed wavenumber decrease and increase, during cooling and heating respectively, of the *ν*_s_CH_2_ band arising from the membrane’s phospholipid acyl chains, indicates a membrane lipid phase transition in both the control and in the presence of Mg(ClO_4_)_2_.

The first derivative of the *ν*_s_CH_2_ curve as a function of temperature was plotted to determine the lipid phase transition temperatures. The phase transition observed upon cooling in Fig. 3a, referred to as the solidification transition (T_s_) relates to the transition from a disordered membrane fluid state (liquid-crystalline phase) to an ordered rigid state (gel phase) upon cooling. The pellet exposed to 2 M Mg(ClO_4_)_2_ exhibited a T_s_ value slightly lower than for the Tris control (4.2 and 7.8 °C, respectively). However, *ν*_s_CH_2_ values overlapped for control and 2 M Mg(ClO_4_)_2_ pellets at temperatures higher than 20 °C and lower than −10 °C, thus indicating maintenance of the lipid cell membrane organization in the presence of Mg(ClO_4_)_2_. The melting transition observed upon heating (T_m_) in Fig. 3b refers to the inverse process by which the membrane transitions from an ordered gel phase to the more fluid liquid-crystalline phase. The exposure to 2 M Mg(ClO_4_)_2_ induced a smaller difference in T_m_ compared to the control cells (7.5 and 8.4 °C respectively).

The hysteresis between T_m_ and T_s_ for control cells and those exposed to 2 M Mg(ClO_4_)_2_ was 0.6 and 3.3 °C respectively. This may suggest some degree of lateral phase separation induced by the presence of Mg(ClO_4_)_2_.

## Discussion

The low temperature limit for life is elusive, multifaceted, and poorly characterized. While kinetic and thermodynamic arguments proposed that there is no low temperature limit for metabolism^2^, empirical observations of cellular activity significantly lower than −25 °C are yet to be made. Clarke et al., suggested that the onset of intracellular vitrification enforces a low temperature limit for life as cellular processes become inhibited due to high internal viscosity. As low temperature aqueous environments are expected to be found elsewhere in the solar system, such as the Martian subsurface or the icy moons, it is important to understand how the low temperature limit for life affects the habitability of such extreme environments.

It was shown that cryoprotectants such as glycerol, either produced in vivo or provided exogenously, can depress intracellular vitrification^7^. However, the ability of inorganic salts, known to be present in extraterrestrial settings, to depress intracellular vitrification was not known. Therefore, in this study, we examined the ability of Mg(ClO_4_)_2_ to depress intracellular vitrification, as perchlorate salts have been identified to be ubiquitous components of the Martian environment, and are hypothesized to be required at molar concentrations to maintain liquid water in Martian subsurface environments^10^.

Here, we have shown that 2.5 M Mg(ClO_4_)_2_ lowers the peak vitrification temperature of *Bacillus subtilis* from −21 °C, to −83 °C, the lowest recorded intracellular vitrification temperature in the current literature. With Martian subglacial environments expected to be ~−70 °C these results show that Mg(ClO_4_)_2_ can, in principle, allow cells to remain in a potentially active, liquid-like state in deep subzero Martian environments. Life in such a cold environment would be exhibiting metabolic turnovers on the scale of millions of years^2^ due to high intracellular viscosity, which while slow, could still imply the potential for active maintenance processes if cells were trapped for a billion or more years. This observation demonstrates that natural inorganic eutectic molecules can potentially alter the low temperature limit for life.

Using FTIR, we also confirmed that the vitrification peaks observed in the DSC did not correspond to lipid phase transitions, giving confidence to the interpretation that the minor thermal events observed are indeed due to intracellular vitrification. In terms of membrane structure, we observed that *B. subtilis* cells retained their overall cellular structure post freeze-thaw and perchlorate incubation (Supplementary Fig. 1). However, Live/Dead staining (assay outlined in Supplementary Methods) showed that these intact cells exhibited variable membrane permeability post freeze-thaw in the presence of Mg(ClO_4_)_2,_ as seen in Supplementary Fig. 2. Therefore, despite the increased membrane permeability due to freeze-thaw and perchlorate exposure, the *B. subtilis* cells have retained sufficient structure to vitrify. Previous research on mimetic membranes showed that lipid vesicles are stable even at high perchlorate concentrations^23,24^ and the FTIR results presented herein revealed minimal changes in the liquid-crystal-to-gel transition temperature of *B. subtilis* cells in 2 M Mg(ClO_4_)_2_. Overlapping *ν*_s_CH_2_ values pre and post membrane transition suggests similar levels of membrane organization in control vs Mg(ClO_4_)_2_ exposed cells. Imaging and fluorescent analysis of *B. subtilis* post freeze-thaw and perchlorate exposure revealed that the cells maintained their cellular ultrastructure. Intermediate levels of staining with PI possibly suggest that the freezing process and incubation in Mg(ClO_4_)_2_ facilitates the entry of PI into the *B. subtilis* cells on a local scale, while the macroscopic membrane structure remains largely intact. Furthermore, the Mg(ClO_4_)_2_ induced hysteresis observed between T_m_ and T_s_ may indicate some degree of lateral phase separation which could increase membrane permeability. However for our results, membrane structure/ permeability is only of importance insofar as that the cells could still vitrify and produce a vitrification signal, with the ultimate degree of membrane structure being of secondary concern beyond that. The observation of altered membrane stability/ permeability may give us insight into what ultimately causes perchlorate-induced cell death. We hypothesize that perchlorate salts may diffuse into cells^25^, effecting cell death by unfolding critical intracellular proteins^26^ as they appear to be the most susceptible targets of perchlorate effects^27^.

Our study leaves some important questions. Due to the technical limitations of the DSC used in this study, it remains unknown what effect further abiotic environmental parameters such as pressure have on the glass transition temperature of cells. Understanding how pressure and ions act in concert to ultimately affect intracellular vitrification will be important for assessing the habitability and potential for preservation in cold subsurface environments. Additionally, it is unknown how vitrification would affect cells in an environment that cools over geological time, however, a dependence of freezing and vitrification on cooling rates has recently been observed in binary perchlorate solutions^17^. Furthermore, this study used *B. subtilis*, and while it is a resilient bacterium, it would be important to explore whether known psychrophilic and halophilic adaptations can modulate the glass transition temperature without external factors. In particular, it would be important to determine the combined effects of biological responses to low temperatures and salt stress and the extracellular ionic environment in ultimately affecting the process of intracellular vitrification. Due to the lethal concentrations of Mg(ClO_4_)_2_ used in this study it is unlikely that our observed changes to vitrification are caused by a stress response mounted by *B. subtilis*. Additionally, if a stress response was imparting an effect one would have expected to have seen it in cells exposed to 0.3 M Mg(ClO_4_)_2_ as this is the growth limit for *B. subtilis*. As such it would be interesting to explore the effect of salt-in halophilic biological adaptations in combination with extracellular ions in affecting intracellular vitrification.

Another challenge to life at low temperatures is biomolecular stability. Proteins exhibit a cold denaturation point^28^ which may further limit the potential for active biochemistry at deep sub-zero temperatures. The cold unfolding of proteins is typically not a problem for extant life as ice formation can prohibit their unfolding^29^, but in conditions where ice formation is inhibited, cold unfolding becomes a considerable challenge^30,31^ which life may have developed strategies to avoid^32^. The chaotropic nature of many deep eutectic compounds would also create challenges for life which required these compounds to avoid freezing. For example, perchlorate salts are known to be highly deleterious to protein structure and function^33,34^, with limited data exploring the effects of perchlorate salts on the cold-unfolding of proteins^35^. This terra-incognito is further compounded by considering the combined effects of high pressures and chaotropic salts on the cold unfolding of proteins. To fully understand the ability of eutectic compounds to affect the low temperature limit for life, it will need to be determined whether such compounds can extend cellular metabolic activity to lower temperatures, beyond the vitrification limit which would be experienced in their absence. In essence, much is yet to be discovered about the limits to life in extreme environments.

## Methods

### Materials

*Bacillus subtilis* DSM 10 was obtained from DSMZ, Mg(ClO_4_)_2_, meat extract, and peptone were obtained from Sigma-Aldrich. LIVE/DEAD^®^ BacLight^TM^ Bacterial Viability Kit (L13152) (Invitrogen) was obtained from Thermo Fisher.

### *Bacillus subtilis* Culturing and Preparation

*B. subtilis* was cultured overnight at 30 °C, 100 rpm in 50 mL of liquid media (5 g/L peptone, 3 g/L meat extract, pH 7.8) in 250 mL conical flasks. Cells were then centrifuged at 10,000 × *g*, 4 °C for 10 min. The pellets were then combined and further washed, twice in 0.1 M Tris buffer (pH 7.8) and once in deionized water, with cells pelleted at 10,000 × *g*, 4 °C for 10 min between washes. The pellet was centrifuged once more in a microfuge at 10,000 × *g*, at ambient temperature for 1 min to remove any excess solution from the pellet. Cells were harvested at this point for perchlorate-free measurements. For perchlorate-containing experiments, the cell pellet was resuspended in a solution of Mg(ClO_4_)_2_ at various concentrations (0.3, 0.6, 1, 2, 2.5 M) for one hour at room temperature before being pelleted and centrifuged twice at 10,000 × *g*, 4 °C for 10 min, with a final microfuge spin at 10,000 × *g*, ambient temperature for 1 min to again remove excess solution from the cell pellet. 0.3 M was chosen as the lowest concentration as it was the highest known tolerable concentration for *B. subtilis* growth^22^. Post-incubation, the cells were spun down twice in a microfuge and then the pellet was stored on ice.

### Cellular vitrification point determination/differential scanning calorimetry

Differential scanning calorimetry was used to measure the vitrification point of *B. subtilis* cell pellets. Experiments were performed on a Diamond DSC (Perkin Elmer LLC; Norwalk, CT, USA) equipped with a liquid nitrogen cooling accessory (CryoFill; Perkin Elmer). For DSC scans, ~20–30 mg of wet *B. subtilis* cell pellet, prepared as described above, were added to a DSC pan and sealed. Perchlorate free measurements were taken by removing a portion of the cell pellet post washing in deionized water. The DSC scan rate was set to run from 10 to −150 °C at 10 °C/min, held at −150 °C for 1 min, then heated from −150 to 20 °C at 10 °C/min. The vitrification point of *B. subtilis* was recorded during the heating ramp of the DSC scan. For analysis, Pyris software (V13.3.1, Perkin Elmer) was used. The first derivative of the heat flow thermogram was taken and smoothed. The onset, peak, and end temperatures of cellular vitrification were recorded during the heating phase as T_onset_, T_peak_, and T_end_ respectively, as shown in Fig. 1b, d.

### Membrane lipid phase transition/FTIR spectroscopy

The membrane lipid phase transition of whole *B. subtilis* cells during freezing and heating was studied using Fourier Transform Infrared (FTIR) spectroscopy. Measurements were carried out on a Nicolet Magna 750 FTIR spectrometer (Thermo Fisher Scientific; Madison, WI, USA) equipped with a mercury/cadmium/telluride (MCT) detector and a variable temperature sample holder (Specac Ldt.; Orpington, Kent, UK), as described by Gautier et al.^36^. For FTIR measurements, a small amount of *B. subtilis* cell pellet, washed in Tris buffer (0.1 M, pH 7.8) or exposed to 2 M Mg(ClO_4_)_2_ solution, was tightly sandwiched between two calcium fluoride (CaF_2_) windows (ISP Optics; Riga, Latvia). Temperature was decreased from 50 °C to −50 °C at a rate of 2 °C.min^−1^ then heated at the same rate to 50 °C. Infrared absorption spectra acquisition was performed throughout the temperature scan by the Omnic software (version 7.1, Thermo Fisher Scientific; Madison, WI, USA). Each spectrum was obtained from 32 co-added scans collected in the mid-IR region between 4000 and 900 cm^−1^, every 45 s at a 4 cm^−1^ resolution. Spectra analyses were performed using the ASpIR software (Infrared Spectra Acquisition and Processing, INRAE; Palaiseau, France). Membrane lipid phase behavior was monitored by measuring the position of the symmetric CH_2_ stretching vibration band (*ν*_s_CH_2_) located around 2850 cm^−1^ and arising from the lipid acyl chains of the cytoplasmic membrane. To determine specific peak locations from each spectrum, second-order derivatives were calculated and smoothed following a 7-point Savitzky-Golay algorithm. The *ν*_s_CH_2_ peak, at 2850 cm^−1^, was plotted as a function of temperature. The *ν*_s_CH_2_ plots were fitted with a curve based on an asymmetric sigmoid transition function and the first-order derivative was calculated. Its maximum was taken as the lipid phase transition temperature following freezing (T_s_, solidification following freezing, and T_m_, melting following heating, in °C).

### Statistics and reproducibility

For DSC analysis, *B. subtilis* cell pellets from multiple 50 ml cultures were combined into one large pellet. 20–30 mg of this cell pellet were taken per measurement, constituting one replicate. The number of replicates for DSC were as follows, 0 M: 17, 0.3 M: 16, 0.6 M: 20, 1.0 M: 18, 2.0 M: 16, 2.5 M: 16. For the FTIR measurement, a small portion of one *B. subtilis* pellet incubated in 0 M and 2 M Mg(ClO_4_)_2_, was taken and measured.

### Supplementary information

Supplementary information

Description of Additional Supplementary Files

Supplementary Data

## Data Availability

The source data for Figs. 1, 2, 3, and Supplementary Fig. 2 can be found in Supplementary Data file
