# Supplementary material for: Preservation of Bacillus subtilis’ cellular liquid state at deep sub-zero temperatures in perchlorate brines
Source: Commun Biol. 2024 May 16;7:588. doi: 10.1038/s42003-024-06277-4 (PMC11099114; doi:10.1038/s42003-024-06277-4)
Supplement: Supplementary file 1 — Supplementary information [file 42003_2024_6277_MOESM1_ESM.pdf]

Supplementary information

Preservation of cellular liquid state at deep sub-zero temperatures in perchlorate brines

Supplementary methods

Membrane Integrity/ Fluorescence Microscopy and Spectroscopy:

To confirm that *B. subtilis* cells could retain their cellular macrostructure post perchlorate exposure and freeze-thaw, the LIVE/DEAD® BacLight™ Bacterial Viability Kit (L13152) (Invitrogen) was used to investigate the membrane integrity of *B. subtilis* in assayed concentrations of $Mg(ClO_4)_2$. Overnight cultures of *B. subtilis* were pelleted at 10,000 g, 4 °C for 10 minutes, then resuspended in either 0.1 M Tris buffer, pH 7.8, 70 % ethanol, or $Mg(ClO_4)_2$ solutions (0.3, 0.6, 1, 2, 2.5 M). Cells were incubated in these solutions for 1 h, before being frozen in a -80°C freezer for 2 hours and then thawed at room temperature as a proxy for the incubation, cooling, and heating cycle they would have experienced in the differential scanning calorimetry experiments. The cells were then pelleted and resuspended twice, before finally being resuspended in 0.1 M Tris buffer, pH 7.8, so that the assay pH was constant and could not affect the fluorescence measurements. For fluorescence spectroscopy the cell density was adjusted to ~0.1 OD_{670}. A 2X stock of LIVE/DEAD® dye was prepared by dissolving the STYO9 and propidium iodide (PI) in 5 mL of deionized water. For fluorescence measurements, an equal volume of cell suspension was mixed with the 2X dye stock giving final working concentrations of 6 µM SYTO9 and 30 µM propidium iodide and cells were left to incubate in the dark for 15 minutes. Cells which were initially incubated in Tris buffer and 70 % ethanol acted as live and dead controls, respectively, with which to compare the perchlorate containing conditions.

Fluorescence microscopy was performed with a Nikon ECLIPSE Ti microscope, using a 60x/1.4 oil immersion objective. For SYTO9, EGFP: Chroma set 49002 (Excitation 470/40, dichroic 495LP, Emission 525/50) was used. For PI, DSRed: Chroma set 49005 (Excitation 545/30, dichroic 570LP, Emission 620/60) was used. Images were processed with ImageJ software. The SYTO9 and PI signals are presented as blue and red respectively, with regions of overlap presenting as magenta.

For fluorescent microplate measurements, a standard curve was created in which the Tris and 70 % ethanol conditions represented 100 % and 0 % membrane integrity respectively, with 90:10, 50:50, and 10:90 mixtures of the two representing 90 %, 50 %, and 10 % membrane integrity, respectively. The fluorescence intensity of the SYTO9 (green) and PI (red) signal was determined with a CLARIOstar Plus fluorimeter (BMG Labtech) equipped with a dual linear variable monochromator. Samples were excited at 485 nm and emission was recorded at 530 nm for STYO9 and 630 nm for PI. Sample measurements were repeated in quadruplicate. The ratio of these signals (G/R ratio) was determined and used to extrapolate the membrane integrity percentage of the perchlorate containing conditions.

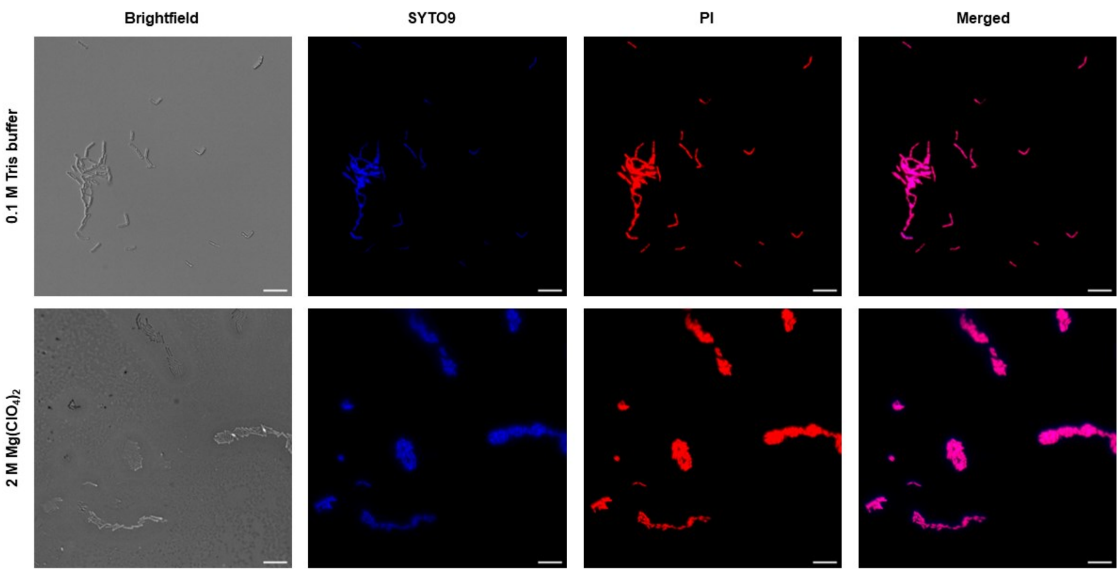

Supplementary Figure 1: *Bacillus subtilis* cells post freeze-thaw. Representative images of *B. subtilis* cells incubated in 0.1 M Tris buffer and 2 M $Mg(ClO_4)_2$. Live/Dead stains are shown as blue (SYTO9), and red (PI), with regions of overlap denoted by magenta. Scale bars are 10 μm.

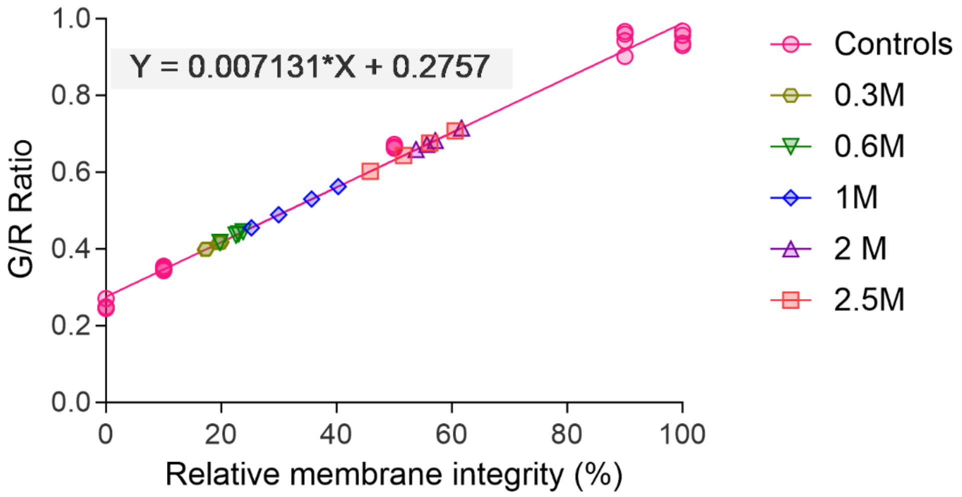

Supplementary Figure 2: Relative membrane integrity of *Bacillus subtilis* across $Mg(ClO_4)_2$ concentrations. A standard curve (pink line) of *Bacillus subtilis* membrane integrity created by plotting the SYTO9/ PI ratio (G/R Ratio, arbitrary units) of cells in mixtures of 0.1 M Tris buffer and 70 % ethanol. Tris buffer: 70 % ethanol-treated ratios of 100:0, 90:10, 50:50, 10:90, and 0:100 represented 100, 90, 50, 10, and 0 % membrane integrity respectively (pink circles). Membrane integrity of cells post incubation in 0.3 M (dark green hexagons), 0.6 M (green triangles), 1 M (blue diamonds), 2 M (purple triangles), and 2.5 M (red squares) $Mg(ClO_4)_2$ are shown. The equation for the slope is provided.
