# Supplementary material for: Preservation of Bacillus subtilis’ cellular liquid state at deep sub-zero temperatures in perchlorate brines
Source: Commun Biol. 2024 May 16;7:588. doi: 10.1038/s42003-024-06277-4 (PMC11099114; doi:10.1038/s42003-024-06277-4)
Supplement: Supplementary file 2 — Description of Additional Supplementary Files [file 42003_2024_6277_MOESM2_ESM.pdf]

# Description of Additional Supplementary Files

**File name:** Supplementary Data

**Description**: The source data for Figures 1,2,3, and Supplementary Figure 2.
